# Unpacking the key components of a programme to improve the timeliness of hip-fracture care: a mixed-methods case study

**DOI:** 10.1186/s13049-015-0171-6

**Published:** 2015-11-09

**Authors:** Pamela Mazzocato, Maria Unbeck, Mattias Elg, Olof Gustaf Sköldenberg, Johan Thor

**Affiliations:** Medical Management Centre, the Department for Learning, Informatics, Ethics and Management, Tomtebodavägen 18A, Karolinska Institutet, SE-17177 Stockholm, Sweden; Department of Clinical Sciences, Danderyd Hospital, Karolinska Institutet, Division of Orthopaedics, SE-18288 Stockholm, Sweden; Department of Management and Engineering, Linköping University, SE-581 83 Linköping, Sweden; Vinnvård Fellow of Improvement Science, The Jönköping Academy for Improvement of Health and Welfare, Jönköping University, P O Box 1026, SE-551 11 Jönköping, Sweden

**Keywords:** Hip fracture, Process improvement, Quality improvement, Timeliness, Case study, Statistical process control charts

## Abstract

**Background:**

Delay to surgery for patients with hip fracture is associated with higher incidence of post-operative complications, prolonged recovery and length of stay, and increased mortality. Therefore, many health care organisations launch improvement programmes to reduce the wait for surgery. The heterogeneous application of similar methods, and the multifaceted nature of the interventions, constrain the understanding of which method works, when, and how. In complex acute care settings, another concern is how changes for one patient group influence the care for other groups. We therefore set out to analyse how multiple components of hip-fracture improvement efforts aimed to reduce the time to surgery influenced that time both for hip-fracture patients and for other acute surgical orthopaedic inpatients.

**Methods:**

This study is an observational mixed-methods single case study of improvement efforts at a Swedish acute care hospital, which triangulates control chart analysis of process performance data over a five year period with interview, document, and non-participant observation data.

**Results:**

The improvement efforts led to an increase in the monthly percentage of hip-fracture patients operated within 24 h of admission from an average of 47 % to 83 %, with performance predictably ranging between 67 % and 98 % if the process continues unchanged. Meanwhile, no significant changes in lead time to surgery for other acute surgical orthopaedic inpatients were observed. Interview data indicated that multiple intervention components contributed to making the process more reliable. The triangulation of qualitative and quantitative data, however, indicated that key changes that improved performance were the creation of a process improvement team and having an experienced clinician coordinate demand and supply of surgical services daily and enhance pre-operative patient preparation.

**Conclusions:**

Timeliness of surgery for patients with hip fracture in a complex hospital setting can be substantially improved without displacing other patient groups, by involving staff in improvement efforts and actively managing acute surgical procedures.

## Background

Hip fracture is a major cause of morbidity and suffering, with Sweden and the other Scandinavian countries exhibiting among the highest incidence of hip fracture in the world [1, 2]. Many patients die in the aftermath of their hip fracture. A Swedish study exploring changes in hip-fracture care in Stockholm during 1998–2007 found, despite a decrease in mortality over the 10-years period, high mortality rates overall. Of all patients, 12.2 % died within 4 months after discharge and 9.0 % after 4 to 12 months. Among patients aged 85 years or more, 33.4 % died in hospital or within one year [3].

Delays to surgery are associated with more post-operative complications, prolonged recovery and length of stay, and even with increased mortality [4–7]. Conversely, earlier surgery (whether within 24, 48, or 72 h), is associated with lower risk of death and complications, e.g. postoperative pneumonia or pressure ulcer [7]. Complicating the treatment many patients with hip fracture are elderly and frail with three or more co-morbidities [3]. Thus, hip-fracture treatment requires the coordinated efforts of multiple units, specialties and professionals. When this coordination fails, patients may suffer from avoidable delays and suffering [8]. Therefore, in recent years, many health care organisations have launched improvement programmes to achieve better coordinated care processes with shorter time to surgery. Methods commonly reported in the literature include integrated care pathways (ICPs) [9], pre-operative fast-track [10–12], and orthogeriatric models [9, 13, 14].

ICPs, also termed critical pathways, care paths, or care maps, are defined as written tools detailing recommended steps in the care of patients with a specific condition and describing the expected progress of the patient [15]. Despite this general definition, their application often entails heterogeneous approaches [9]. One common challenge is that the responsibility for managing the care for older adults presenting with a hip fracture is not allocated to a single individual; instead, multiple professionals with specific narrower scopes of responsibilities are involved, with scant coordination of efforts. Through ICPs, the clinical care process from hospital admission to discharge is supported by standard protocols based on evidence-based guidelines [9]. ICPs for hip fracture patients are associated with beneficial effects on short term outcomes, e.g. fewer post-operative complications, while their impact on short and long term mortality is less clear [9, 16].

Fast-track strategies promote rapid transition from the emergency department (ED) to an orthopaedic ward [10–12, 17, 18]. When combined with ICPs, fast-tracks can reduce patients’ length of stay, whereas the impact on mortality rates is unclear [10–12]. Implemented alone, fast-track systems have shortened patients’ length of stay in the ED [17, 18].

Orthogeriatric models involve collaboration between the orthopaedic surgeon and the geriatrician [9, 13, 14]. Patients are admitted to an orthopaedic ward but both the orthopaedic surgeon and the geriatrician share responsibility for the care of the patient [14]. While the heterogeneity of this approach complicates the interpretation of the findings, orthogeriatric models have been associated with reduced short and long term mortality [9, 14].

Despite the positive results associated with ICPs, fast-track, and orthogeriatric models applied to hip-fracture care, it is not possible to define one best organisational model of care for this patient group [9]. The heterogeneous application of similar methods, and the multifaceted nature of the interventions, constrain generalizations about which method works, when, and how. To gain a deeper understanding of what works, research needs to better disentangle what is actually being implemented [19, 20] and how the multiple components of improvement interventions contribute, or do not, to improved operational performance. Moreover, as such interventions often target one patient group, whose care takes place in parallel with the care for many other patient groups, it is important to understand whether improvement is due to increased efficiency in the care process (“win-win”) or simply to re-allocation of capacity from one patient group to another (“win-lose”) [21].

We therefore set out to analyse how multiple components of hip fracture improvement efforts aimed to reduce the time to surgery influenced that time both for hip-fracture patients and for other acute surgical orthopaedic inpatients.

## Methods

### Design

This is an observational mixed-methods single case study [22] of process improvement efforts at the Danderyd Hospital, based on non-participant observations, interviews, documents, and administrative data including the time from arrival to the hospital to surgery.

### Setting

The Danderyd Hospital is a public university hospital located in the Stockholm metropolitan area. In 2003, the National Board of Health and Welfare in Sweden issued a recommendation for hip-fracture care that prescribed surgical treatment within 24 h from admission [23]. In 2009, to encourage adherence to these guidelines, the Stockholm County Council, the regional health service commissioner, introduced a financial incentive to hospitals requiring (among other things) that 80 % of their hip-fracture patients be operated within 24 h of admission. With more than 600 hip-fracture patients per year, this patient group constituted the largest group of acute patients hospitalized at the orthopaedic department, with a total of some 3700 patients admitted annually to its four inpatient wards. The department had made several attempts to improve hip-fracture care since the 1990’s. Despite these efforts, in 2008 only 49 % of patients reached the operating table within 24 h. Therefore, in June 2009 the hospital management reignited improvement efforts. A multidisciplinary team was formed, including professionals involved in the treatment of hip-fracture patients from all involved units, to further improve the existing process and, thereby, to reduce patients’ wait for surgery. The specific aim of the improvement initiative was that 80 % of hip-fracture surgeries should start within 24 h of hospital admission.

### Data collection and analysis

Data collection and analysis was organized in a qualitative and a quantitative phase as described below.

#### Development of a case description

The first author conducted 19 semi-structured interviews with members of the improvement team, other clinicians, managers, and improvement advisors. Interviews focused on four main areas: characteristics and needs of hip-fracture patients; organisation of the care process (both before and during the intervention) including goals, routines, roles and responsibilities, coordination mechanisms, and process challenges; changes planned and implemented; and effects of the changes as experienced by staff members. She also collected about 40 documents, including meeting notes from each process improvement meeting from June 2009 through December 2012, hip-fracture care protocols and job descriptions. These documents provided information mainly on the intervention’s components and process problems identified by the improvement team. The first author also conducted non-participant observation by shadowing caregivers within the different units as well as three patients from arrival to the start of surgery. She focused on patient, work, and information flows, with the aim to understand how the process worked in practice. She also attended improvement team meetings to gain a deeper understanding of how the intervention was designed and carried out. Data collection lasted from June 2009 through December 2012.

The group of authors included both members internal and external to the case organization. PM, ME and JT had no explicit roles in the organizational change but rather acted as external researchers. MU and OGS were part of the improvement team and their role in the qualitative analysis was to validate the case description.

Taking a stepwise approach, the first author reviewed the qualitative data to develop a case description. Documents were organized in chronological order in an Excel file to reconstruct the change process. Data collected through interviews, documents, and observations were organized and coded in NVivo 8 software (QSR International Pty Ltd, 2008) to characterize a) process problems (before and during the redesign), b) the actual changes carried out, and c) the effects of changes as reported by staff members.

#### Quantitative analysis of performance

Data on the lead time to surgery (from arrival to the hospital until the start of surgery, in hours) was collected for hip-fracture patients and other acute surgical orthopaedic inpatients for the period January 2008 through December 2012. This period covers about one and a half year prior to, and three and a half years after, the start of improvement efforts.

To assess the impact of process changes we used statistical process control (SPC) charts to analyse patterns of performance over time [24–26]. The control chart supports separation of common-cause and special-cause variation. Common-cause variation is inherent in a process, and depends on chance. Special-cause variation represents non-random change which is due to some influence not previously part of the process, such as improvement interventions.

Different control charts are applicable in different situations [27]. In the present study, a p-chart, where p stands for proportion, was appropriate for plotting the proportion of patients per month operated within 24 h from arrival to the hospital. The p-chart is useful for routine monitoring of a binary outcome where the sample size varies at each data point [25]. Features of an SPC chart include a central line (CL), i.e. the average value of all observations, and upper (UCL) and lower control limits (LCL). Control limits reflect the range of variation in the data and were set, as customary, at three standard deviations, or 3σ, from the central line [27, 28]. Two decisions rules were used to detect special-cause variations: any single data point outside the 3σ limits or nine consecutive data points on the same side of the central line [25, 27]. The SPC analysis is further described in Appendix 1.

We used one-way independent analysis of variance (ANOVA) to assess whether different intervention components influenced the lead time to surgery for hip fracture and for the total acute orthopaedic patient flows. To do this, we divided the time-to-surgery data into periods from baseline by when key intervention components were introduced. ANOVA was also undertaken to assess negative as well as positive spill over effects on the lead time to surgery for other acute orthopaedic inpatients. When significant changes were detected, post hoc analysis was conducted using pair-wise comparisons with LSD (least significant difference). The significance level was set at p =0.05.

We performed chi-square analysis for patient age, gender, and diagnosis to control for case mix variation in the hip-fracture patient group for baseline and the various intervention components. Statistical analyses were performed using MINITAB 16.2.1 software (Minitab Inc.).

## Results

The case study findings are presented in four main sections. First, we describe the care process prior to improvement efforts as well as the main challenges faced in the management of hip-fracture care; second, we present the key changes implemented and how staff perceived them; third, we show the operational performance for hip-fracture care and its relationship to key intervention components; fourth, we report the lead time to surgery for all other acute orthopaedic inpatients to identify possible spill-over effects.

### Care process prior to the intervention

About 90 % of hip-fracture patients arrived at the orthopaedic section of the ED via ambulance. There, patients with a suspected hip fracture were triaged by a registered nurse (RN). Nursing staff started the diagnostic and treatment process according to local clinical guidelines. A physician then assessed the patient further and sent a referral for an x-ray examination. When needed, the physician also sent a referral to a specialist in internal medicine or in cardiology. The physician preliminarily requested a bed on one of the three acute orthopaedic wards. At the radiology unit, patients were examined in order of arrival, i.e. patients with hip fracture were not prioritized before other acute patients. After the examination, if a fracture was confirmed, the patient was transferred directly to the ward. When the physician then received the x-ray report, he/she completed the administrative admission work, including adding the medication list, and registering the patient on the acute surgery list.

On the orthopaedic ward, nursing staff prepared the patient for the surgery according to an individualized prescription and written standard procedures. Pre-operative assessment and optimization of the patient in preparation for surgery was mainly a responsibility of anaesthesiologists. In some cases, patients were also referred to a specialist in internal medicine or in cardiology. Pre-operative assessment was often delayed due to problems in the previous steps, e.g. lacking test results, or delayed patient record transcription. Delays also occurred because of competing demands on the time of internists, cardiologists, and anaesthesiologists from multiple patient groups.

At the central surgical unit, two operating rooms (ORs) were dedicated to acute orthopaedic surgeries during day-time on weekdays, serving both inpatient and outpatient cases. Capacity for hip-fracture patients was constrained by the fact that only one of the two ORs was equipped with the fracture table needed to operate this patient group. During off-hour shifts, resources at the central surgical unit were shared with the general surgery and urology services. This could cause dispute between specialties over how to prioritize patients. During office-hours, surgery planning was mainly a responsibility of the two RN coordinators at the central surgical unit (the anaesthesiology and operating theatre RN), in collaboration with the most senior orthopaedic physician available. The fact that there was no physician with the overarching responsibility to plan acute surgeries, that different surgeons were scheduled every day at the central surgical unit, and that different surgeons had different opinions on which patients to prioritize, often resulted in delays due to uncertainty about which patient should be operated, by whom, how, and when. Moreover, scheduling of surgeries for the following day was often carried out at the end of the day-shift. Thus, if patients arrived during evening and night, scheduling did not take place until the next day. When it was time to operate the patient, the anaesthesiology RN coordinator at the central surgical unit contacted a RN colleague on the ward, who then administered the pre-operative medication. The patient was transferred to the pre-operative room at the central surgical unit, a room just outside the OR, where an anaesthesiologist, who served several ORs, initiated the anaesthesia. The patient was then moved into the OR, where the operating team prepared the patient for surgery. Surgery then started upon the surgeon’s arrival. Delays here could also occur due to problems in the previous phases of the care process, such as a patient was not fully optimized for surgery or was not ready from the ward, the medication list was lacking, or the anaesthesiologist was lacking information on the type of surgery.

### Changes implemented to improve the timeliness of hip- fracture surgery

The formation of the improvement team to reach the 24 h goal, which included health care professionals with key roles in care of hip-fracture patients, focused many stakeholders’ attention on hip-fracture patients. In turn, as expressed by staff in the interviews, the focus on the target increased awareness among staff of the (clinical and organizational) importance of providing timely care to this elderly and frail patient group. In the section below we describe the main changes introduced after the constitution of the improvement team in June 2009, in chronological order.

#### Centralize and clarify the responsibility for planning acute surgeries (anaesthesia and surgery start phase)

In August 2009, the responsibility for planning acute orthopaedic surgeries during office hours was, for the first time, assigned to a consultant orthopaedist. The consultant was also the newly appointed head of the trauma unit at the orthopaedic department. She worked in close collaboration with the two RN coordinators at the central surgical unit to: i) plan which surgeries should be performed at the central surgical unit, and which should be scheduled as day-surgeries at the outpatient unit; ii) choose and plan an appropriate surgical procedure; iii) assign cases to different orthopaedic surgeons to match the procedure with the required competence level; iv) continually monitor patient inflow and (re-)schedule acute surgeries; and, v) communicate and coordinate with colleagues working on evening and night shifts to choose and plan an appropriate surgical procedure. Written standard procedures were developed to standardize surgery planning; these included trying to routinely schedule a hip-fracture case as the first procedure each morning with a focus on operating these patients within 24 h. Staff at the central surgical unit described how centralized planning reduced uncertainty about which patient should be operated, when, by whom, and how. This change also contributed to reducing the likelihood that hip fracture cases were postponed when other acute cases emerged.

#### Checklist at the wards

In October 2009, the orthopaedic wards introduced a checklist outlining the steps to be taken to get hip fracture patients ready for surgery. Staff reported that the checklist made the content and timing of work more explicit. The positive effects of a more standardized care process were also observed by staff at the central surgical unit, who reported that they found patients to be prepared for surgery in a more timely and correct manner than before.

#### Ambulance fast-track for hip-fracture patients with no other suspected fractures or acute diseases

In January 2010, one of three ambulance operators who provided transport to the hospital was selected to test an ambulance fast-track. Patients who the ambulance staff suspected had a hip fracture (according to certain explicit criteria) received certain treatment, e.g. pain medication and oxygen, and were taken by that staff directly to the radiology unit, thus by-passing the ED. Patients with signs of multiple fractures, or other complicating conditions, as well as patients arriving on their own, were excluded and seen first in the ED as usual. All hip-fracture patients were now admitted to the same orthopaedic ward, and not to multiple wards as previously done. On the ward, an RN responsible for admitting hip-fracture patients took necessary tests according to protocol. The physician on service in the ED left to make an admission examination on the ward, registered the patient in the acute surgery list, and filed the medications list. To clarify roles and responsibilities in this new process, the improvement team developed new written standard procedures, a checklist with inclusion and exclusion criteria for ambulance staff, and a job description for the admitting RN. Staff reported that the ambulance fast-track helped improve patients’ experience, by reducing (often painful) movements and enabling faster transfer to a ward bed.

#### Orthopaedic acute day-surgeries moved to the outpatient unit (anaesthesia and surgery start phase)

In June 2010, orthopaedic day surgery procedures were moved from the central surgical unit to the outpatient unit. Two ORs could thus now be dedicated to surgery for acute orthopaedic inpatients. Furthermore, both rooms were now equipped with the fracture table required to operate hip-fracture patients. According to staff members interviewed, this made it easier to schedule acute inpatient surgeries during day-time. Concurrently, a policy was introduced to avoid performing acute surgeries later than 21.00 (except cases that could not wait for clinical reasons), whereas before, many acute surgeries were performed around the clock.

#### Standard procedures to support timely patient optimization

In June 2010, standard procedures started to be formalized to support the ED physician in starting the patient optimization process as soon as possible, including clear criteria for when to consult a specialist in internal medicine, in cardiology or in anaesthesiology. Clear goals and expectations were developed for anaesthesiologists to make sure that at least one hip-fracture patient was ready for surgery each morning. For this last component, we were unable to identify a clear-cut implementation date. Nevertheless, staff reported that more often there was a hip-fracture patient ready for surgery in the morning after the introduction of the new procedures.

#### Four extra beds at the orthopaedic ward

In February 2011, the hospital management added four extra orthopaedic inpatient beds based on considerations not restricted to the improvement initiative described here but related to a general need for more inpatient beds. These four extra beds were reserved for hip-fracture patients.

#### Expand ambulance fast-track to all ambulance operators

In March 2011, the hospital extended the ambulance fast-track to include all three ambulance operators, based on favourable experiences from the pilot test. Consequently, the proportion of patients admitted via the ambulance fast-track rose from 18 % (January 2010-February 2011) to 46 % (March 2011-December 2012).

#### Specialist in internal medicine

In August 2011, a specialist in internal medicine was employed to work during office hours at the orthopaedic department to improve (non-surgical) care for patients in need of specialized medical care, such as those suffering from multiple co-morbidities. The assignment was to assess patients, make any adjustments in their treatment in preparation, and facilitate the coordination among the multiple care professionals, e.g. RNs, the orthopaedic surgeon in charge, and the anaesthesiologist, during the pre and post-operative care process. Staff reported coordination among care professionals in the pre-operative phase to be improved, which in turn contributed to reduce the time to surgery.

#### Flow coordinator at the central surgical unit

In a pilot test between May and December 2012, an RN coordinator planned acute surgeries during weekends. This was in addition to the RN coordinators who worked at the central surgical unit during office hours. Given the positive results observed, this role was kept after the pilot test.

### Performance changes

**Fig. 1:**
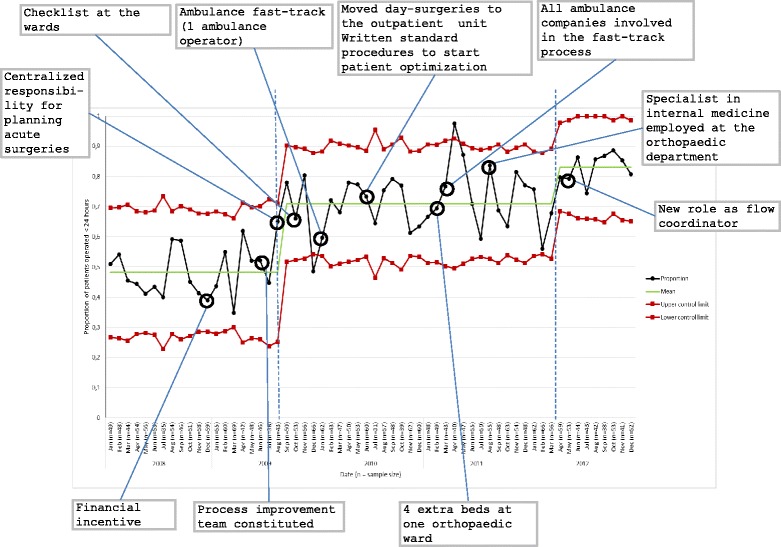
C*onsecutive monthly proportion of hip fracture patients operated within 24 h analysed in a p-chart*

The p-chart (Fig. 1) shows the consecutive monthly proportion of hip-fracture patients undergoing surgery within 24 h. We annotated the chart with the timing of changes for which there was a clear-cut implementation date. The SPC analysis (detailed in Appendix 1) shows that systematic improvements (“shifts”) in process performance occurred at two time points. Until July 2009, the process exhibited a lower average proportion operated within 24 h than in subsequent months. The first systematic improvement in performance occurred two months after the constitution of the improvement team when the consultant orthopaedist assumed the coordinator role and adopted explicit routines for centralized planning of acute surgeries at the central surgical unit. Thus, this first improvement in performance was achieved through changes to the management of the care process without adding resources. A second systematic improvement occurred in April 2012. The starting of this “shift” coincided with the establishment of a flow coordinator out-of-office hours, which thus likely contributed to the new level of performance. On average, the percentage of patients operated within 24 h increased from 47 % before improvement efforts begun (January 2008 – July 2009), to 71 % in the first improvement level (August 2009 – March 2012), and 83 % in the second improvement level (April 2012 – December 2012). This third phase also exhibits a narrowing range of process variation.

During the first improvement phase, two data points fell outside the 3σ limits (one on each side). One of these points followed right after the ambulance fast-track was extended to all ambulance companies. Since these observations occurred in isolation, they did not indicate a sustained shift in performance. There are no indications of significant seasonal variation in the time to surgery. On the contrary, the p-diagram shows three rather stable periods.

**Fig. 2:**
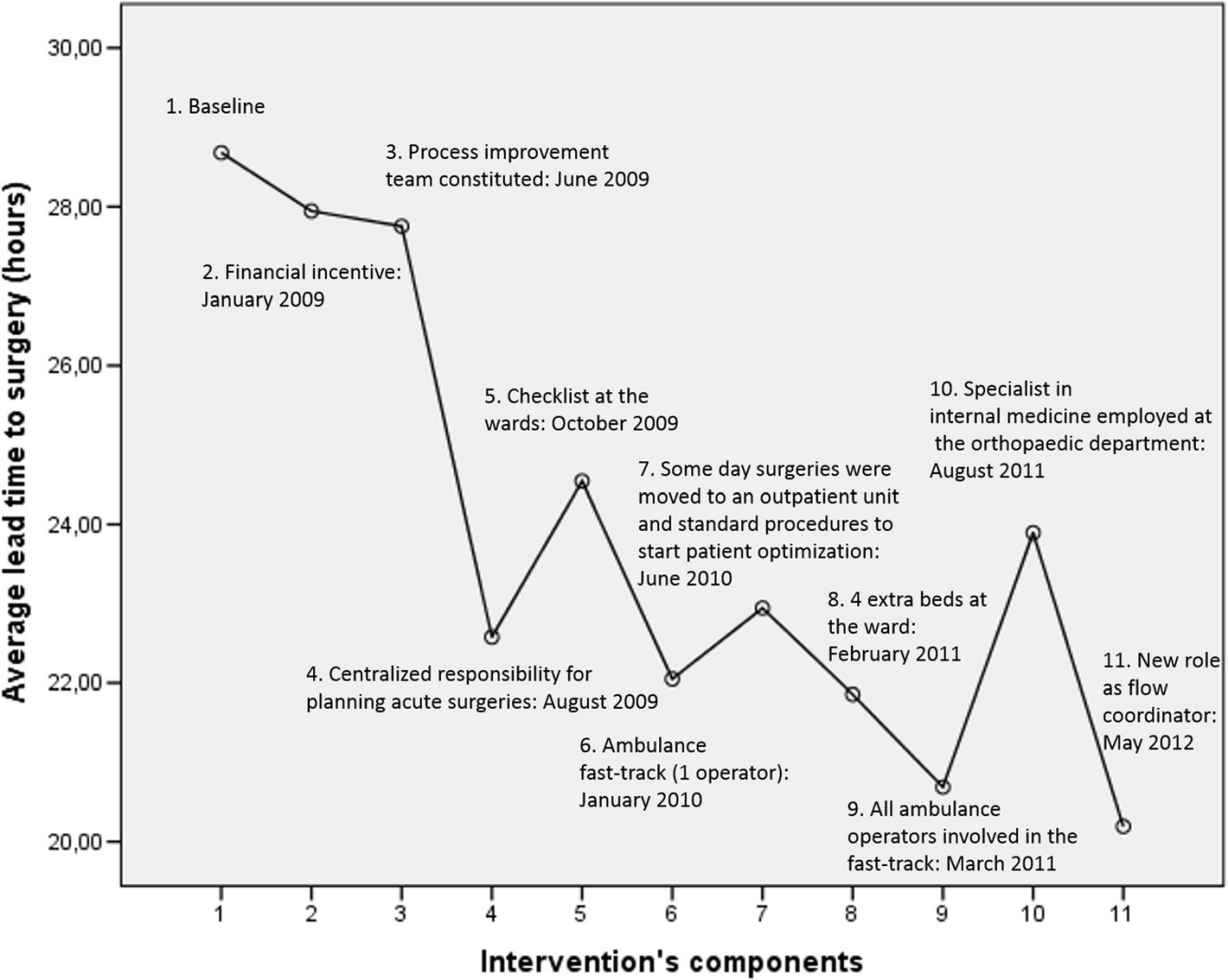
Lead time (hours) to surgery for hip-fracture patients

This general improvement pattern for the care of hip-fracture patients mirrors the ANOVA (Fig. 2) for changes in the average lead time to surgery which coincide with when the hospital introduced different improvement components.

The post hoc analysis, presented in Table 1, confirmed that a statistically significant change in performance occurred after the introduction of a role and routines to centrally plan acute orthopaedic surgery (intervention’s component number 4 in Fig. 2). The difference in average time to surgery between intervention 3 (mean = 27.8 h) and 4 (mean = 22.6 h) was 5.2 h (p = 0.02). No statistically significant improvement in performance was observed when the ambulance fast-track was extended to all ambulance companies (intervention’s component number 9 in Fig. 2). This supports the SPC finding that, the special cause variation observed in April 2011 did not represent a systemic shift in performance, but rather occurred in isolation.

**Table 1:** Post hoc comparisons using least significant difference

	Baseline (1) (*n* = 607)	Financial incentive (2) (*n* = 277)	Process improvement team constituted (3) (*n* = 81)	Centralized responsibility (4) (*n* = 116)	Checklist (5) (*n* = 151)	Ambulance fast track (6) (*n* = 266)	Moved day- surgeries (7) (*n* = 395)	4 extra beds (8) (*n* = 49)	All ambulance fast track (9) (*n* = 244)	Specialist in internal medicine (10) (*n* = 514)	Flow coordinator (11) (*n* = 364)
Baseline (1)	0	0.7 (−1.4; 2.8)	0.9 (−2.5; 4.3)	6.1^*^ (3.2; 9.0)	4.1^*^ (1.5; 6.8)	6.6^*^ (4.5; 8.8)	5.7* (3.9; 7.6)	6.8* (2.5; 11.1)	8.0* (5.8; 10.2)	4.8* (3.1; 6.5)	8.5* (6.6; 10.4)
Financial incentive (2)		0	0.2 (−3.5; 3.8)	5.4^*^ (2.2; 8.6)	3.4^*^ (0.5; 6.3)	5.9^*^ (3.4; 8.4)	5.0^*^ (2.7; 7.3)	6.1^*^ (1.6; 10.6)	7.3^*^ (4.7; 9.8)	4.1^*^ (1.9; 6.2)	7.8^*^ (5.5; 10.1)
Process improvement team constituted (3)			0	5.2^*^ (1.0; 9.4)	3.20 (−0.8; 7.2)	5.7^*^ (2.0; 9.4)	4.8^*^ (1.3; 8.3)	5.9^*^ (0.7; 11.1)	7.1^*^ (3.4; 10.8)	3.9^*^ (0.4; 7.3)	7.6^*^ (4.0; 11.1)
Centralized responsibility (4)				0	−2.0 (−5.5; 1.6)	0.5 (−2.7; 3.7)	−0.4 (−3.4; 2.7)	0.7 (−4.2; 5.6)	1.9 (−1.4; 5.1)	−1.3 (−4.3; 1.7)	2.4 (−0.7; 5.5)
Checklist (5)					0	2.5 (−0.4; 5.4)	1.6 (−1.2; 4.4)	2.7 (−2.1; 7.4)	3.9^*^ (0.9; 6.8)	0.6 (−2.0; 3.3)	4.4^*^ (1.6; 7.1)
Ambulance fast track (6)						0	−0.9 (−3.2; 1.4)	0.2 (−4.3; 4.7)	1.4 (−1.2, 3.9)	−1.8 (−4.0; 0.3)	1.9 (−0.5; 4.2)
Moved day-surgeries (7)							0	1.1 (−3.3; 5.5)	2.3 (−0.1; 4.6)	−1.0 (−2.9; 1.0)	2.8^*^ (0.7; 4.9)
4 extra beds (8)								0	1.2 (−3.4; 5.7)	−2.0 (−6.4; 2.3)	1.7 (−2.7; 6.1)
All ambulance (9)									0	−3.2* (−5.5; −1.0)	0.5 (−1.9; 2.9)
Specialist in internal medicine (10)										0	3.7^*^ (1.7; 5.7)
Flow coordinator (11)											0^*^

Detailed legend: Mean differences (and 95 % confidence intervals) shown. *indicates statistical significance (*p* < 0.05)

The ANOVA indicated a statistically significant decrease in performance after intervention 10 – when the specialist in internal medicine was employed – although this did not correspond to a systematic change in performance as shown in the SPC chart in Fig. 1. The difference in the average time to surgery between intervention 9 (mean = 20.7 h) and 10 (mean = 23.9 h) was −3.2 h (p = 0.01). After this decrease in performance, time to surgery decreased again when the flow coordinator role was extended also to weekends (intervention’s component 11 in Fig. 2). The mean difference between intervention 10 (mean = 23.9 h) and 11 (mean = 20.2 h) was 3.7 h (*p* < 0.01).

There were no changes in the case-mix with regard to age, gender, and diagnosis that could explain changes in the lead time to surgery. The proportion of patients in different age groups (<75, 76–85, >85) did not differ, *χ*^2^ (16, N = 1967) = 24.5, p = 0.08. Furthermore, there were no changes in the proportions of female and male patients, *χ*^2^ (8, N = 1967) = 3.4, *p* = 0.91, or in the different forms of hip fracture (fractures of the femoral neck, the trochanters, or the inter- or subtrochanteric region), *χ*^2^ (16, N = 1967) = 24.5, *p* =0.94.

For other acute surgical orthopaedic inpatients, ANOVA indicates no significant, negative or positive, changes in lead time to their surgery between the intervention’s components (*p* = 0.46).

## Discussion

The improvement efforts investigated in this study led to an increase in the percentage of hip-fracture patients operated within 24 h from an average of 47 % to 83 % and a performance that will predictably range between 67 % and 98 % if the process continues without changes. The key changes that contributed to this improvement were the creation of a process improvement team and the introduction of roles and routines for centralized management of acute orthopaedic surgeries. The improvement observed for hip-fracture patients did not occur at the expense of other patient groups – there were no significant changes in lead time to surgery for other acute surgical orthopaedic inpatients.

### Unpacking the programme’s key components

The long-term and multi-component improvement efforts enabled the case organization to initially reach levels of performance not previously reported in the literature, and then to sustain and further improve this performance over the three and a half years study period. Other studies have reported the percentage of hip-fracture patients operated within 24 h to reach 56 % [12], 58 % [29], and 69 % [10] following similar improvement efforts. Several of the changes made by the case organization have been described previously in the literature as part of fast-track systems [10–12, 17, 18] and ICPs [9, 16], e.g. the definition of procedures based on evidence, timing, and allocation of responsibilities. The contribution of this study lies in how we were able to unpack, through a mixed methods approach, the complex intervention programme and reveal how each component influenced operational performance.

This study shows how an explicit target (such as the 24 h target) to improve access to care triggered process improvement initiatives which reduced delays and unnecessary waiting due to poor process management. Several authors argue, however, that if a focus on achieving targets is not accompanied by the development of better process capability, there is a risk that results will not be sustained [30, 31] since the level of performance is an inherent characteristic of any system [32, 33]. The triangulation of qualitative and quantitative data and methods showed that the development of roles and routines for surgery planning, first only during week-days and office hours and later also on weekends, were key to reaching the target. Previous studies have found the availability of surgical services to affect the timeliness of care for hip-fracture patients [11], but that there is a knowledge gap regarding planning and scheduling procedures for acute surgical services [34]. Our study demonstrates how centralized management of acute orthopaedic services can enable organisations to better match supply with demand by making (the right) resources available for patients when they need them. Active daily planning reduced unnecessary waiting for hip-fracture patients without additional resources while other patient groups, largely dependent on the same resources, did not experience longer time to surgery.

The ambulance fast-track did not appear to influence the ability to reach the target for early surgery. This could have several explanations. First, only 46 % of the patients were admitted via the ambulance fast-track. Second, while all ambulance companies were involved in the fast-track, it took them some time to train their staff and thus the fast-track was implemented in a step-wise manner. Consequently, it took time for the actual effects of the fast-track to play out in practice. While these are all plausible explanations, our findings are consistent with other studies that have shown that fast-track systems can reduce waiting time to admission to the ward [17, 18], but have little [10], if any [12, 17], effect on time to surgery.

According to our qualitative data, several changes may have made the process more reliable. We cannot, however, fully assess how different intervention components influenced and interacted with one another, but the ANOVA indicates that several subsequent intervention components had a cumulative effect on the process performance. To further clarify such questions would require different methodology, including tests of removing discrete process changes or factorial study designs [35], which were not feasible in the current study.

Previous research on improvement interventions for single patient groups, such as ICPs, often neglected the possible “side-effects” on other patient groups [21]. Because multiple patient groups often compete for similar services using the same shared resources, such as an operating room [36], it is important to understand how, like in the case organisation, improvement for one patient group can be achieved without hampering performance for another. Our study shows that it was possible to improve services in the complex environment of acute care without significant side effects in other processes.

### Methodological considerations

Understanding cause and effect relationships when improvement efforts involve multiple intervention components implemented in a complex environment can be challenging [33, 37]. It is particularly challenging in observational studies in which changes are designed and applied by clinicians and managers in an emergent manner rather than specified beforehand by a protocol. We illustrate how a mixed methods design can be used to address this challenge. Qualitative interview and observation data can be used to characterize planned changes and to compare them with the actual changes implemented. Qualitative interview data can also be used to assess the effects of such changes as they are experienced by healthcare professionals. These experiences can then be compared with the effects detected in quantitative data via SPC analysis and post-hoc tests. Using such triangulation, the more indications that a certain change has an effect (and a lack of plausible competing explanations), the stronger the evidence. An example in this study was the effect of centralized planning, where the confluence of evidence was strong. In contrast, the evidence for a negative effect on time to surgery of the employment of a specialist in internal medicine, identified through the post-hoc analysis, was weak as no such effect was confirmed by either SPC or the qualitative analyses. This example is particularly illustrative of the value of combining SPC and traditional statistical analysis, especially when studying long-term improvement efforts and simple pre-post designs may be misleading [38].

In addition to using mixed methods, we employed other strategies to strengthen our study’s internal validity [22]. Data collection followed a case study protocol outlining the purpose of the research and the approach chosen. Key informants reviewed a draft of the case description to validate it.

A single case study design has inherent external validity limitations due to the contextual nature of the knowledge generated. As several organisations around the world are struggling to reach the same goal of timely care delivery for hip-fracture patients, cross-organisational and cross-county comparisons are possible and needed to bridge contextual particularities.

## Conclusions

Timeliness of care for patients with hip fracture can be substantially improved by involving clinicians in systematic improvement efforts and introducing clear routines for managing acute surgical procedures. Appointing an experienced clinician to coordinate demand and supply of surgical services on a daily basis enhanced pre-operative patient preparation and scheduling of surgery. This highlights the importance in a complex hospital setting, which routinely deals with multiple parallel processes, of not only having highly competent clinicians and evidence-based pathways, but also of actively managing care processes.

## Appendix: successive identification of stages in performance

First analysis:

This step was made by checking any trends and significant changes for the whole data set. That means that we calculated one centre line, UCL and LCL based on the whole data set. Traditional formulas for p-diagram are used. We base decision on the following alarm rules: one point outside 3 sigma and 9 points in a row on the same side of the centre line.

The figure below shows this graph.

The analysis reveals that the first period until July 2009 indicates a process that generates lower performance than the other months. So the second step is to treat this period as the starting period and separate it from the rest of the data.

Second analysis:

This second analysis was made by checking any trends and significant changes when separating the starting period from the first improved level. We base decision on the following alarm rules: one point outside 3 sigma and 9 points in a row on the same side of the centre line.

The graph is showed below:

The analysis revels four alarms. First, we see three data points outside the three sigma limits. Since they occur only sporadically we don’t see any motives for changing the control limits based on them. They are only single alarms but do not indicate a stable change. However the fourth alarm point indicates that there is an alarm based on nine points in a row on the same side of the centre line. This interpreted as a systematic change in performance leading to a new improved performance level. Thus the final graph is the one that is showed in the article.

## Abbreviations

CL: central line
ED: emergency department
ICPs: integrated care pathways
LCL: lower control limit
LSD: leas significant difference
OR: operating room
RN: registered nurse
SPC: statistical process control
UCL: upper control limit
